# The Wnt/β-catenin pathway regulates growth and maintenance of colonospheres

**DOI:** 10.1186/1476-4598-9-212

**Published:** 2010-08-06

**Authors:** Shailender S Kanwar, Yingjie Yu, Jyoti Nautiyal, Bhaumik B Patel, Adhip PN Majumdar

**Affiliations:** 1 Department of Internal Medicine, School of Medicine, Wayne State University, Detroit MI, 48201, USA; 2 John D. Dingell V A Medical Center, Detroit MI, 48201, USA; 3 Karmanos Cancer Institute, Detroit MI, 48201, USA

## Abstract

**Background:**

Recent evidence suggests that epithelial cancers, including colorectal cancer are driven by a small sub-population of self-renewing, multi-potent cells termed cancer stem cells (CSCs) which are thought to be responsible for recurrence of cancer. One of the characteristics of CSCs is their ability to form floating spheroids under anchorage-independent conditions in a serum-free defined media. The current investigation was undertaken to examine the role of Wnt/β-catenin pathway in regulating the growth and maintenance of colonospheres. Human colon cancer cells HCT-116 (p53 wild type; *K-ras *mutant), HCT-116 (p53 null; *K-ras *mutant) and HT-29 (p53 mutant) were used.

**Results:**

Colonospheres formed *in vitro *exhibited higher expression of colon CSCs markers LGR5, CD44, CD166 and Musashi-1 along with putative CSC marker EpCAM, compared to the corresponding parental cancer cells and also exhibit the ability to form spheroids under extreme limiting dilution, indicating the predominance of CSCs in colonospheres. Colonospheres formed by HCT-116 cells show over 80% of the cells to be CD44 positive, compared to ≤ 1% in the corresponding parental cells. Additionally, colonospheres showed reduced membrane bound β-catenin but had increased levels of total β-catenin, cyclin-D1 and c-myc and down regulation of axin-1 and phosphorylated β-catenin. Increased expression of β-catenin was associated with a marked transcriptional activation of TCF/LEF. The latter was greatly decreased following down regulation of β-catenin by the corresponding siRNA, leading to a marked reduction in CD44 positive cells as well as colonospheres formation. In contrast, upregulation of c-myc, a down-stream effector of TCF/LEF greatly augmented the formation of colonospheres.

**Conclusion:**

Our data suggest that colonospheres formed by colon cancer cell lines are highly enriched in CSCs and that Wnt/β-catenin pathway plays a critical role in growth and maintenance of colonospheres.

## Background

A growing body of evidence supports the contention that epithelial cancers including the colorectal cancer are diseases driven by a small set of self renewing cells, termed cancer stem cells (CSC) or cancer-initiating cells, that are distinct from the bulk of the cells in the tumor [1]. Initially identified in hematopoietic tumors, CSCs have now been identified and isolated in a variety of solid tumors that include breast, central nervous system, pancreas, skin, head and neck, colon and prostate [2-7]. CSCs share all the fundamental traits of stem cells-self renewal by asymmetric division, reduced proliferation and differentiation and resistance to apoptosis [8]. CSCs are identified by specific surface epitopes, which in the colon include CD44, CD133 and CD166 [9,10]. To select putative colorectal CSCs, a promising combination of three markers- EpCAM, CD44 and CD166 was described by Dalerba *et al. *[11]. Although EpCAM previously being considered as pan-epithelial marker in the normal human colon, its frequent expression in CSCs in breast, colon, pancreas and prostate tumors suggests that this surface epitope could be a putative marker for CSCs, particularly in human colon cancer-derived cell lines [12]. More recently Lgr5, Musashi-1 and aldehyde-dehydrogenase 1 (ALDH-1) have been added to the list of stem cell markers for colon cancer [13-15].

One of the recently reported characteristics of tumor derived CSCs is that they can be grown to form spherical colonies *in vitro*, when plated in limited numbers under anchorage-independent conditions in a serum-free defined media supplemented with growth factors [9]. With the objectives to promoting *in vitro *expansions of CSCs, methods have been developed to grow and study them in sphere-forming assays as reported for neurospheres [16,17], mammospheres [18,19] and colonospheres [13,20]. Using this approach, we and others have identified and/expanded colon CSCs by generating colonospheres from colon cancer cell lines [10,21-24]. However, little is known about the signaling events that regulate the growth and maintenance of colonospheres.

Different signaling pathways such as Wnt, Hedgehog, Notch and Bmi have been implicated in various cellular processes during development that include differentiation, migration and proliferation [25-27]. Recent studies have reported the pivotal role of Wnt/β-catenin signaling pathway in the regulation of epithelial stem cell self renewal [28,29]. In contrast, dysregulation of Wnt/β-catenin signaling has been implicated in colon carcinogenesis [30,31]. However, the regulatory role of Wnt/β-catenin signaling in the maintenance and growth of colonospheres still remains elusive. The current investigation was, therefore, undertaken to study the *in vitro *expansion of colonospheres that display the characteristics of CSCs and to delineate the role of Wnt/β-catenin pathway in regulating the growth and maintenance of colonospheres using three different human colon cancer cells: HCT-116 (p53 wild-type; *K-ras *mutant), HCT-116 (p53 null; *K-ras *mutant) and HT-29 (p53 mutant, *K-ras *wild-type).

## Results

### Generation and characterization of colonospheres

**Figure 1:**
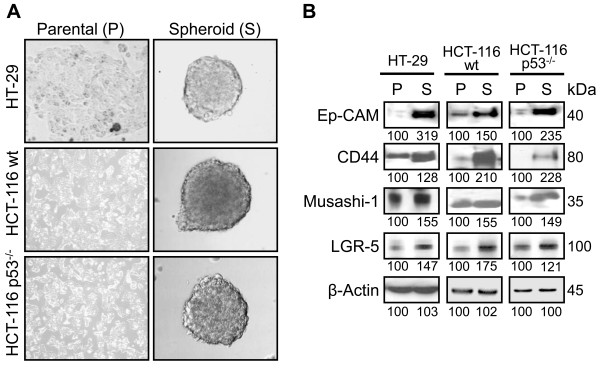
**CSC markers in colon cancer cells and spheroids/colonospheres**. **A**. Representative photographs showing different colon cancer cells (HT-29, HCT-116 wt & p53-null) in adherent condition and colonospheres formed by the respective cell lines. **B**. The comparative expression of different CSC markers in parental colon cancer cells (referred to as 'P') and the cells from corresponding spheroids (referred to as 'S'). The numbers represent percent of corresponding control normalized to β-actin.

**Figure 2:**
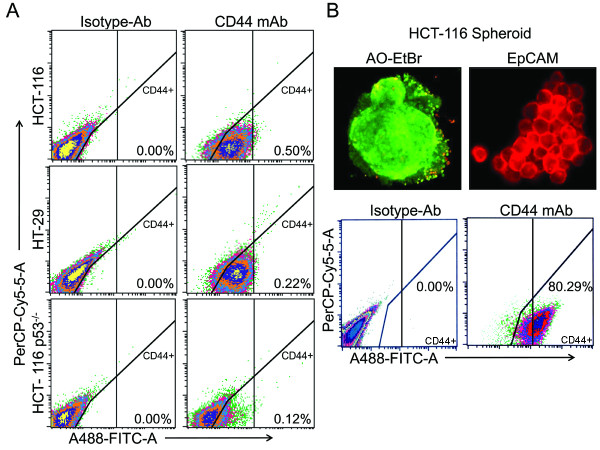
**Spheroids/colonospheres display increased levels of CSC markers**. **A**. Representative scattered plots of flow cytometry analyses of CD44 positive cells in different colon cancer cells: Figures in the left panel depict the parental colon cancer cells stained with PerCP-Cy5 mouse IgG2b (Isotype), whereas those in the right panel, show the cells treated with CD44 conjugated PerCP-Cy5 mouse anti-human antibodies. Values represent percentage of cells that are positive for CD44-PerCP-Cy5. **B**. Photomicrographs showing the integrity and viability of colonospheres (HCT-116) using acridine orange-ethidium bromide (AO-EtBr) staining and immunofluorescence staining depicting the membrane bound expression of putative CSC marker EpCAM (Upper Panel); and flow cytometric analysis showing the proportion of CD44 positive cells in HCT-116 colonospheres-derived cells. Corresponding isotype for CD44 antibodies is shown (Lower Panel).

Recently, we reported that FOLFOX-surviving colon cancer cells that are enriched in CSCs grow in large round, unattached floating spheroid colonies (termed colonospheres) when cultured in chemically defined serum-free medium at a relatively low density [22]. In agreement with our previous observation, our current results also show that colon cancer cells, whether they are p53-positive (HCT-116 wt) or p53-negative (HCT-116p53^-/- ^and HT-29) form spheroid colonies in a chemically defined media (Figure 1A). The spheroids formed by these cells showed higher levels of EpCAM (also known as epithelial specific antigen or ESA) along with colon CSC markers-CD44, Musashi-1 and LGR-5 than the corresponding parental cells (Figure 1B). When the proportion of the CD44 positive cells was determined by flow cytometry in parental cells and colonospheres, they were found to be ≤ 1% in parental cell lines (Figure 2A), as opposed to more than 80% observed in colonospheres (Figure 2B). The results suggest that colonospheres formed by the colon cancer cells are enriched in CSCs. Indeed, when the colonospheres formed by HCT-116 cells were subjected to immunofluorescence staining for EpCAM, we observed bright EpCAM staining on the cell surface of each cell of colonospheres indicating the presence of CSCs in colonosphere (Figure 2B). However, since EpCAM is also expressed in normal human epithelial cells as well as in gastrointestinal carcinomas, we carried out the next set of experiments to validate and quantitate the presence of functional CSCs in colonospheres (within the confines of an *in vitro *system). We determined the frequency of sphere-forming cells by performing an extreme limiting dilution analysis (ELDA) for parental and colonospheres-derived HCT-116 cells. The frequency to form spheroids was found to be 5.5 fold higher for cells derived from colonospheres than those from the parent cell line (Table 1).

**Table 1:** Extreme limiting dilution analysis of colonospheres forming frequency of HCT-116 cells, transfected with non-targeted (vector) or β-catenin siRNA and by colonospheres-derived cells

Number of cells/well	Number of wells plated	Number of wells showing colonospheres		
		
		Colonospheres-derived cells	Parental/Vector transfected	Parental/β-catenin siRNA transfected
1000	24	24	24	24
100	24	24	24	10
10	24	24	14	2
1	24	14	4	0

Sphere forming frequency (95% CI)		1/2 (1/3-1/2)	1/11 (1/17-1/7)	1/171 (1/287-1/102)

*P *value		<< 0.0001		

Data are pooled from three independent experiments for each. CI, confidence interval

**Figure 3:**
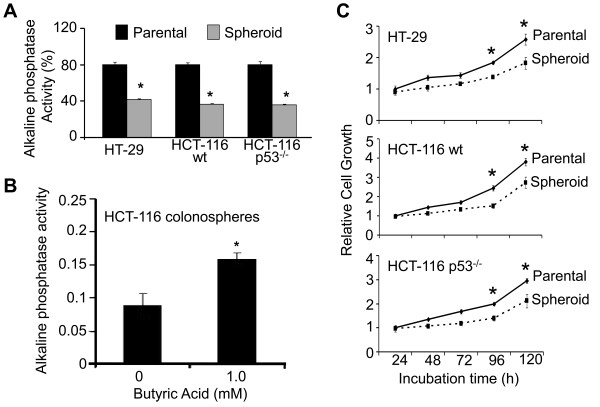
**Differentiation and proliferation potential of parental colon cancer cells and the corresponding spheroids/colonospheres**. **A**. Spheroids show decreased alkaline phosphatase activity compared to parental colon cancer cells. **B**. Induction of alkaline phosphatase activity in the spheroids in response to Na-butyrate. **C**. Changes in cellular growth of parental cell lines and colonospheres over the period of 5 days as determined by MTT assay. Each value represents mean ± SD of six observations. *p < 0.001, compared to the corresponding parental cells or control.

Like normal stem cells, CSCs are also poorly differentiated and proliferate slowly [32]. To determine the extent of differentiation of colon CSC, colonospheres formed by HCT-116wt, HCT-116p53-null and HT-29 cells and the corresponding parental cells were assayed for alkaline phosphatase activity. The results revealed a 50-55% reduction in alkaline phosphatase activity in colonospheres when compared with their corresponding parental cells (Figure 3A). Further, to determine whether cells in colonospheres can be induced to differentiate, they were incubated for 24 h with 1 mM sodium butyrate, a known differentiation inducing agent [33]. Sodium butyrate caused a 2-fold increase in alkaline phosphatase activity in colonospheres, compared to the controls (Figure 3B). Since colonospheres are composed of cells that display the characteristics of CSCs, we examined their proliferative potential. When cells from colonospheres formed by HCT-116 (wt and p53-null) and HT-29 were analyzed for cell growth by MTT assay over a period of 5 days (120 h), we found the growth to remain 30-40% lower over the corresponding parental cell lines (Figure 3C).

**Figure 4:**
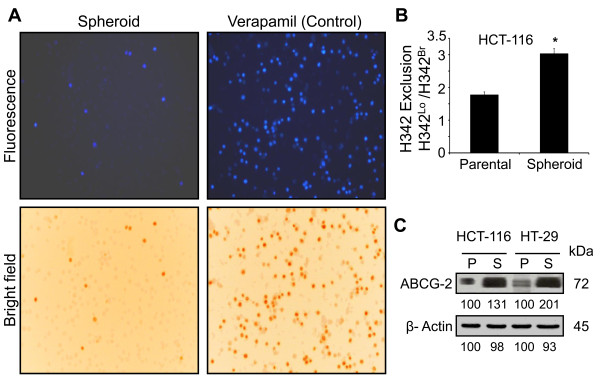
**Spheroids/colonospheres-derived cells show increased dye-efflux**. **A**. Photomicrographs showing exclusion of Hoechst-33342 (H342) by HCT-116 colonospheres-derived cells in the absence or presence of verapamil, an inhibitor of membrane ABCG2 transporter protein (positive control). Propidium Iodide was used to detect dead cells. **B**. The quantitaion of flow cytometry analysis of H342 dye exclusion by HCT-116 parental and colonospheres-derived cells. **C**. Western blot showing changes in the levels of ABCG-2 in HT-29 and HCT-116 wt parental (P) and colonospheres-derived cells (S). The numbers represent percent of corresponding control normalized to β-actin. *p < 0.01 compared to the corresponding parental cells.

CSCs are known to be resistant to chemotherapy because of increased drug efflux capacity [34]. To determine whether cells in colonospheres that are enriched in CSCs would also show increased exclusion of drugs, the cells from HCT-116 colonospheres were subjected to fluorescence microscopy and flow cytometry analysis for Hoechst 33342 dye exclusion [35]. A marked 80% increase in dye exclusion was observed for cells in colonospheres of HCT-116 cells, compared to the parental cells (Figure 4A, B). This was associated with increased expression of ABC transporter protein ABCG2 (Figure 4C), a member of the superfamily of ATP-binding cassette (ABC) transporters whose primary function is to transport various molecules across the intra- and extra-cellular membranes [34].

### The Wnt/β-catenin pathway is activated in colonospheres

**Figure 5:**
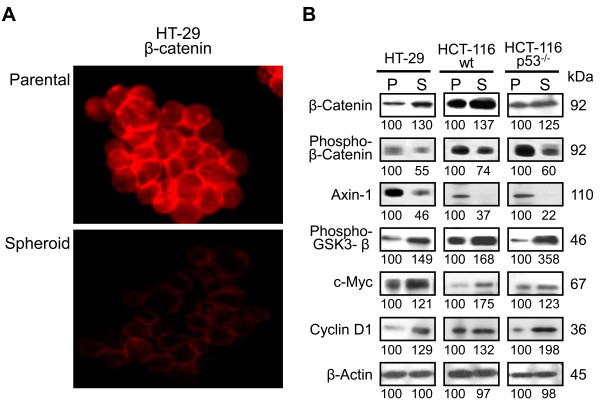
**Activation of Wnt/β-catenin signaling pathway in spheroids/colonospheres**. **A**. Representative photomicrographs of HT-29 parental and colonospheres-derived cells depicting the membrane localization of β-catenin by immunofluorescence staining. **B**. The comparative expression of different members of Wnt/β-catenin signaling pathway in parental cells (referred to as 'P') and colonospheres-derived cells (referred to as 'S'). The Wnt/β-catenin signaling is constitutively activated in colonosphere-derived cells, but not in parental cells as shown by the expression of various proteins: β-catenin, phosphorylated β-catenin, axin-1, phosphorylated GSK-3-β along with important downstream targets: c-myc and cyclin-D1. The numbers represent percent of corresponding control normalized to β-actin.

The Wnt/β-catenin signaling pathway is known to be activated in many malignancies, including colorectal cancer [36]. This signaling pathway also plays a critical role in regulating the proliferation of CSCs [37]. To determine the involvement of Wnt/β-catenin signaling pathway in maintaining the growth and maintenance of colonospheres, the next set of experiments were carried out. The immunofluorescence studies revealed a markedly higher staining of membrane bound β-catenin in HT-29 parental cells, when compared with cells from the corresponding colonospheres (Figure 5A). Next, we compared the expression of several members of Wnt/β-catenin pathway and its downstream targets in the parental monolayer cultures with those of colonospheres. Western blot analysis revealed a markedly higher expression of total β-catenin, phospho-GSK-3β and their downstream effector c-myc and cyclin-D1 in colonospheres from all three cell lines, compared to the corresponding parental cells (Figure 5B). In contrast, the levels of phospho-β-catenin and axin-1 were found to be considerably lower in colonospheres compared to the corresponding parental cells (Figure 5B). Taken together, the results suggest that Wnt/β-catenin signaling is intricately involved in the growth and maintenance of colonospheres.

### β-catenin is important for the generation of colonospheres

**Figure 6:**
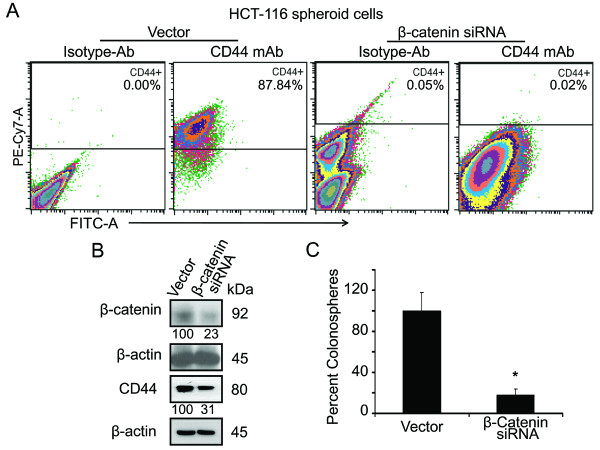
**Downregulation of β-catenin results in reduction of CD44 positive cells in spheroids/colonospheres and inhibits regeneration of colonospheres**. **A**. Flow cytometric analysis shows CD44 positive cells in HCT-116 colonospheres following 72 hrs of transfection with β-catenin siRNA or the vector; corresponding isotypes are also shown, values represent percentage of cells that are positive for CD44-PE-Cy7. **B**. Western blot showing downregulation of β-catenin and CD44 following 72 hrs of transfection of β-catenin-siRNA, compared to the vector-control in HCT-116 colonospheres-derived cells. **C**. Colonosphere formation assay shows decreased regeneration of colonospheres by β-catenin-knockdown HCT-116 colonospheres cells. The numbers represent percent of corresponding control normalized to β-actin. *p < 0.001, compared to the corresponding vector-treated controls

**Figure 7:**
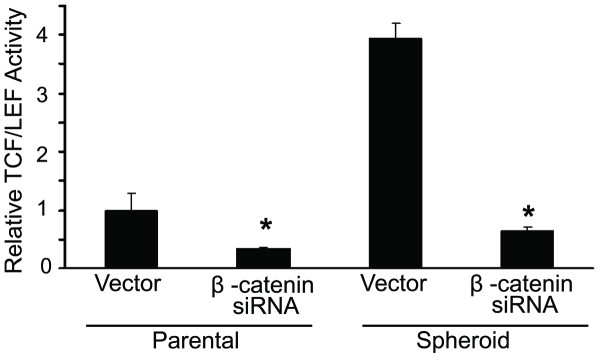
**Downregulation of β-catenin inhibits transcriptional activity of TCF/LEF in parental colon cancer cells and the corresponding spheroids/colonospheres**. TCF/LEF transcription activity in HCT-116 wt parental and colonosphere-derived cells is downregulated in the corresponding β-catenin transfected cells. *p < 0.001, compared to the corresponding vector-treated controls

To demonstrate whether β-catenin plays a key role in the generation of colonospheres, we transfected HCT-116 (wt) cells with β-catenin-siRNA or the corresponding vector (NT-siRNA) to knock-down β-catenin and determined their sphere-forming frequency by performing an ELDA (Extreme Limiting Dilution Analysis). The β-catenin knock-down in HCT-116 cells revealed a 15.5 fold decrease in the number of sphere-forming cells, compared to the parental cells (Table1). Since sphere-forming cells are highly enriched in CSCs that are thought to be critically involved in the formation of colonospheres, it is anticipated that knock-down of β-catenin in colonospheres-derived cells would reduce CSCs leading to reduction in colonosphere formation. Indeed, our flow cytometric analysis revealed that knock-down of β-catenin in colonosphere-derived HCT-116 cells by the corresponding siRNA lead to approximately 87% reduction in the proportion of CD44 positive cells, when compared with the vector-transfected controls (Figure 6A). This observation was further confirmed by Western-blot analysis which showed that a 77% downregulation of β-catenin was accompanied by a marked 70% reduction in the levels of CD44 (Figure 6B). Further, to demonstrate that β-catenin is required for self-renewal of CSCs in colonospheres, regeneration or secondary colonospheres formation assay was carried out following transfection with β-catenin-siRNA or the corresponding vector (NT-siRNA). The downregulation of β-catenin resulted in an 80% reduction in secondary colonospheres formation (Figure 6C). Downregulation of β-catenin also resulted in a marked reduction in Wnt/β-catenin signaling, as evidenced by the significant 60% and 75% decreased transcriptional activity of TCF/LEF in HCT-116 parental cells and colonospheres, respectively, when compared with the corresponding controls (Figure 7).

**Figure 8:**
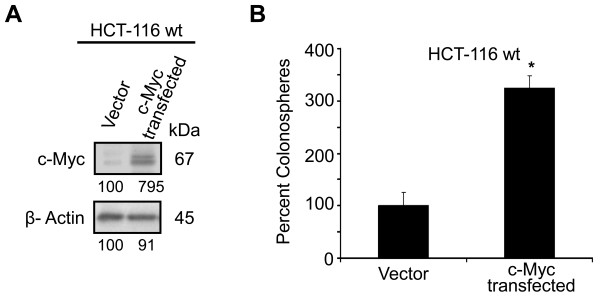
**Upregulation of c-myc enhances colonospheres/spheroid formation**. **A**. Western blot showing changes in c-myc levels in the HCT-116 wt cells following transfections of PCMV6-vector or pCMV6-Neo vector containing human cDNA clone for c-myc gene. **B**. Colonospheres formation for HCT-116 wt parental cells overexpressing c-myc. Each value represents mean ± SD of 3-4 observations. The numbers represent percent of corresponding control normalized to β-actin. *p < 0.001, compared to the corresponding vector-treated control.

To further determine the regulatory role of Wnt/β-catenin signaling in colonospheres formation, the next experiment was carried out to examine whether upregulation of c-myc, a downstream target effector gene of Wnt/β-catenin pathway, in colon cancer cells would augment formation of colonospheres. Western-blot analysis revealed that 3 days following transfection of cDNA for c-myc to HCT-116 cells caused a 7-8 fold increase in the levels of c-myc, when compared with vector-transfected controls (Figure 8A). This was accompanied by a 2.5-fold increase in colonospheres formation by HCT-116 cells, when compared with the vector-transfected controls (Figure 8B).

## Discussion

Recent studies have suggested that many epithelial cancers, including colon cancer arise from a small sub-population of CSCs through their oncogenic transformations [38-40]. There is a strong evidence that colon cancer-derived cell lines represent the tumors from which they are originally isolated [41] and can develop structures similar to those found in the original tissue [42], suggesting the presence of CSCs in colon cancer-derived cell lines. Much of the advance on CSCs comes from the assays that have used FACS technology to isolate and enrich for CSCs from primary tumors, and then testing their propagation in immune-deficient mice [20,43]. Such assays are cumbersome and expensive, and do not readily enable either the characterization of CSCs or their functional properties. However, unlike the primary tumor, the cell lines represent a valuable resource that can be used repeatedly over time, and can be readily characterized by various CSCs assays. There are clearly many advantages to working with CSCs derived from cell lines, both for the possible development of novel targets for drug development, as well as for the possible development of relatively high-throughput drug-response assays for CSCs.

In the present work, using an approach to culture cells that display markers and characteristics of CSCs, we show that spheroid cultures (or colonospheres) generated from a limited number of human colon cancer-derived cell lines are enriched for cells that express colonic CSC markers. Widely accepted general characteristics of "stemness" include the expression of putative stem cell antigens, reduced cell cycle progression, and limited functional differentiation or dedifferentiation under the influence of the microenvironment [10,44]. Currently, colon CSCs are defined by their expression of cell surface markers, such as Musashi, LGR-5, CD44, CD166, etc [11,13-15]. Colonospheres generated under present conditions from the three different colon cancer lines show increased expression of colon CSCs markers along with pan-epithelial marker EpCAM, when compared with the corresponding parental cells. Additionally, the evidence for the presence of functional CSCs in colonospheres came from the ELDA that revealed a marked rise in frequency of sphere-forming ability of cells in colonospheres. Since under extreme limited dilution and in serum-free conditions (stem cell media), only stem-like cells are believed to divide to form spheroids, our observation of formation of colonospheres by a very small number of cells not only suggests the presence of functional CSCs in colon cancer derived cell line but also indicate the enrichment of CSCs in colonospheres [13,20,45].

CSCs have been shown to divide very slow and possess limited differentiation potential [10,36]. The ratio between CSCs and differentiated cells has been suggested to depend on the rate of differentiation relative to the turnover rate of CSCs [46]. In view of this, it is reasonable to suggest that lower the propensity to differentiate greater the proportion of CSCs. Recent reports have also demonstrated low functional differentiation potential of colon cancer cell lines that contain a high proportion of CSCs [10]. Our current data demonstrate that colonospheres are poorly differentiated as evidenced by a marked reduction in alkaline phosphatase activity. Moreover, our observation that colonospheres also show increased H33342 efflux capabilities, accompanied by increased expression of ABC transporter protein ABCG2 suggests that they possess the ability to exclude drugs, a trait that has been demonstrated for CSCs [47,48]. These observations and the fact that over 80% of colonospheres-derived cells are CD44 positive, a widely accepted colon CSC marker, and that the cells of colonospheres also show staining of EpCAM, which is believed to be a putative CSC marker in colon cancer cell lines, strongly indicate that colonospheres generated under present conditions are mainly composed of CSCs.

Increasing evidence suggests that many signaling pathways that are classically associated with cancer, including Wnt, Hedgehog, Notch and Bmi, may also regulate normal stem cell development. One particular interesting pathway that has also been shown to regulate both self-renewal of stem cell and oncogenesis in different organs is the Wnt signaling pathway [36]. Wnt proteins regulate development in several organisms and contribute to cancer when dysregulated through the activation of β-catenin (a downstream activator of the Wnt signaling pathway). The canonical Wnt/β-catenin pathway has previously been reported to play a role in hematopoietic, mammary and brain stem cell functions [28,49,50]. The Wnt pathway is also a major regulator of stem cells in gastrointestinal system as identification of many different Wnt/β-catenin target genes have been implicated in intestinal stem cell proliferation and carcinogenesis [37]. The best stemness-specific target of Wnt/β-catenin signaling known today is Lgr5 [51], while c-myc and cyclin-D1 are the other Wnt targets associated with proliferation of progenitor cells [29,30]. Results derived from our current investigation suggest that Wnt/β-catenin pathway plays a critical role in regulating the growth and maintenance of colonospheres. Initial support for this inference comes from the observations that there is a marked loss of membrane bound β-catenin in colonosphere cells. Further, colonospheres formed by colon cancer HCT-116 (wt or p53^-/-^) and HT-29 show a marked reduction in the expression of phosphorylated form of β-catenin and axin-1 and increased levels of total β-catenin (non-phosphorylated form) and phosphorylated form of GSK-3β, compared to the corresponding controls. Moreover, to inactivate Wnt/β-catenin pathway by GSK-3β, axin is required for phosphorylation of β-catenin [30]. The observed decrease in expression of axin-1 and β-catenin phosphorylation, therefore, suggests that the increased level of total β-catenin is due to the inhibition of β-catenin degradation. Increased levels of β-catenin, following translocation into the nucleus, is likely to cause induction of the TCF family of transcription factors leading to increased synthesis of several proteins that are critically involved in cellular growth [52]. Indeed, our observation that colonospheres from HCT-116 wt and HT-29 cells shows higher levels of c-myc and cyclin-D1, compared to the corresponding parental cells, supports the contention that Wnt/β-catenin signaling plays a critical role in the formation of colonospheres. These findings were further corroborated by the observation that downregulation of β-catenin in the parent cell lines decreases the number of sphere-forming cells as demonstrated by ELDA. Furthermore, during serial passaging of colonospheres, downregulation of β-catenin causes reduction in CD44 positive cells and inhibition in transcriptional activation of TCF/LEF resulting in suppression of regeneration of secondary colonospheres formation. In contrast, overexpression of c-myc is associated with increased colonospheres formation. Taken together, the results strongly suggest that Wnt/β-catenin pathway has a pivotal role in the genesis, maintenance and expansion of CSCs enriched spheroid cultures.

In conclusion, our data demonstrate that colonospheres formed by colon cancer cells are predominantly composed of cells displaying the characteristics of CSCs. Wnt/β-catenin signaling plays a pivotal role in the growth and maintenance of colonospheres in that downregulation of β-catenin that decreases TCF/LEF transcriptional activity resulting in reduction of CD44 positive cells leading to a marked suppression of colonosphere formation and regeneration. In contrast, overexpression of c-myc, a downstream effector of Wnt/β-catenin signaling, reverses the situation. We suggest that the colonosphere formation technique is a useful *in vitro *method to enrich cultures for CSCs or as a surrogate for tumor formation. The data presented in this communication also provide a basis for identification of distinctive molecular pathway(s) for self-renewal of CSCs and for the growth and maintenance of colonospheres.

## Methods

### Cell lines and culture conditions

The human colon cancer cells HCT-116 (p53 wild type; *K-ras *mutant), HCT-116 (p53 null; *K-ras *mutant) and HT-29 (p53 mutant; *K-ras *wt) were obtained from the American Type Culture Collection (ATCC, Rockville, MD). Cells were maintained in Dulbecco's modified Eagle medium (DMEM; 4.5 g/L d-glucose) supplemented with 10% FBS and 1% antibiotic/antimycotic in tissue culture flasks in a humidified incubator at 37°C in an atmosphere of 95% air and 5% carbon dioxide. The medium was changed twice a week, and the cells were passaged using 0.05% trypsin/EDTA.

### Antibodies

Antibodies used for immunofluorescence, or Western blotting were as follows: rabbit anti-LGR5/GPR49 (Abgent, San Diego, CA), mouse anti-β-actin (Chemicon International, Billerica, MA), mouse anti-CD44 (Abcam Inc., Cambridge, MA), rabbit anti-Musashi-1 (Imgenex, San Diego, CA), mouse anti-cyclin-D1, rabbit anti-ABCG2, mouse anti-β-catenin (Santa Cruz Biotechnology Inc., Santa Cruz, CA); rabbit anti-c-myc, mouse anti-EpCAM, mouse anti-CD44, rabbit anti-axin1, rabbit anti-phospho-β-catenin, Cell Signaling, Danvers, MA) and FITC-conjugated mouse-IgG2b (Sigma-Aldrich Inc, St Louis, MO). Mouse anti-human PerCP-Cy5 IgG2b (isotype) or CD44 conjugated antibodies (Santa Cruz Biotechnology Inc., Santa Cruz, CA) and mouse anti-human PE-Cy7 IgG2b (isotype) or CD44 conjugated antibodies (BD Pharmingen) were used for flow cytometry.

### Western blotting

For all Western blot analyses, protein was harvested from adherent cells and colonospheres. Cell lysates were prepared by homogenizing the cells in lysis buffer as previously described [22]. Quantification of the proteins was carried out using a modified Bradford assay (Bio-Rad Laboratories). Protein samples for Western blotting were prepared by boiling after the addition of denaturing sample buffer. Proteins were separated using SDS-PAGE on an 8% or 15% gel and transferred to a polyvinylidene difluoride membrane (Millipore) by electroblotting. Antibodies were diluted in TBS and 0.1% (v/v) Tween with 5% nonfat dry milk after 1 h of protein blocking in the absence of antibodies. Membranes were incubated at 4°C overnight with the primary antibody, subsequently washed and incubated with the appropriate horseradish peroxidase-conjugated secondary antibody (Amersham Biosciences) for 1 h at room temperature. Membranes were again washed, and protein bands were visualized using a commercially available enhanced chemiluminescence kit (Amersham Biosciences). When appropriate, membranes were incubated in stripping solution for 30 min at 65°C, washed, and re-probed with a second primary antibody for verification of loading control.

### Formation of colonospheres

The ability of cell lines to form spheres in suspension was evaluated as described by Liu *et al. *[25], with slight modifications. Briefly, primary colonospheres were generated by incubating the limited number of parental HCT-116 (p53 wild-type; *K-ras *mutant), HCT-116 (p53 null; *K-ras *mutant) and HT-29 (p53 mutant; *K-ras *wild-type) cells at a concentration of 100 cells per 200 μL in serum-free stem cell medium (SCM) containing DMEM/F12 (1:1) supplemented with B27 (Life Technologies, Gaithersburg, MD), 20 ng/ml EGF (Sigma, St Louis, MO), 10 ng/ml fibroblast growth factor (Sigma), and antibiotic-anti-mycotic in 24-well ultra low-attachment plates (Corning Inc, Lowell, MA) for 10 days. The colonospheres formed at the end of the incubation period were centrifuged (1000 rpm), dissociated with 0.05% trypsin/EDTA using a 22 gauge needle and then passed through a 40 μM sieve to obtain single cell suspension, as described by Kakarala *et al. *[19]. The single-cell suspension derived from colonospheres that have undergone 15 or more serial passages were used for all experiments. For spheroids formation, an equal number of cells from adherent cell lines and colonospheres cells were plated at 200 cells/100 μL SCM in each 96 ultra low-attachment well (Corning Inc, Lowell, MA). The colonospheres formed after 5 days were evaluated for their number and size by light microscopy.

### Extreme limiting dilution analysis

Extreme limiting dilution analysis (ELDA) was performed as described by Hu and Smyth [45]. Briefly, single cell suspension obtained from adherent or colonospheres-derived cells were plated at concentration of 1000, 100, 10 cells and 1 cell per 100 μl SCM (24 well for each dilution) in 96-well ultra-low attachment and incubated for 5 days. At the end of 5 days, the number of wells showing formation of colonospheres was counted. The frequency of sphere forming cells in a particular cell type was determined using ELDA webtool at http://bioinf.wehi.edu.au/software/elda.

### Flow Cytometry Analysis

Single cell suspension from parental monolayer cell cultures and colonospheres were subjected to direct immunofluorescence staining followed by flow cytometry analysis according to our standard protocol [22]. Briefly, the cells were harvested and washed with PBS. Half a million cells were suspended in 90 μl of PBS containing 0.5% BSA. After 10 min at room temperature, 10 μl of fluorophore conjugated anti-human CD44 antibody was added and incubated for 30 min in dark at room temperature. The samples were then washed and analyzed using a FACS DiVa (BD, San Jose, CA). The cells stained with mouse IgG2b (isotype-negative control) served as gating control. The proportion of CD44 positive cells was determined on the basis of fluorescence intensity-spectra of CD44-conjugated PE-Cy7 or PerCP-Cy5.

### Immunofluorescence cytochemistry

Single cell suspension, obtained from parental and colonosphere, was washed in PBS and fixed in 2-4% formaldehyde for 10 min at 37 °C. They were washed and re-suspended in 0.5% BSA-PBS (blocking buffer) for 10 min, subsequently incubated in primary antibodies at appropriate dilution for 1 h at room temperature. After rinsing with incubation buffer, the cells were resuspended in fluorophore-conjugated secondary antibodies diluted in incubation buffer according to the manufacturer's recommendations and incubated for 30 minutes at room temperature. The cells were then resuspended in PBS after washing with the incubation buffer. Appropriate aliquot of cells were mounted on glass slides immediately before examining under fluorescence microscope.

### Hoechst 33342 dye exclusion assay

Single cell suspension obtained from parental cell lines and colonospheres were washed with PBS (3 times) and stained with Hoechst 33342 or H342 (5 μg/ml, Sigma-Aldrich Inc, St Louis, MO) for 45 minutes at 37°C in HBSS buffer, vortexing gently every 15 min. As a control, a sample was treated with verapamil (Sigma, 50 μM) for ten minutes at room temperature prior to the addition of H342. The stained cells were collected, washed with PBS and resuspended in 3 ml of PBS containing 2 μg/ml of propidium iodide, and subsequently analyzed by flow cytometer-FACS Vantage SE/DiVa SORP (BD Biosciences, San Jose, CA) with all-digital electronics and octagon- and trigon-shaped detector arrays. Excitation of 100 mW at 488 nm was provided by a 177-G argon ion laser (Spectra-Physics, Mountain View, CA) and 200 mW of all-lines UV (351-365 nm) was provided by an Innova 90-5 argon ion laser (Coherent, Palo Alto, CA). Forward and side laser scatter was detected from 488 nm excitation. H342 and propidium iodide fluorescence from UV excitation was split into "blue" and "red" wavelengths by a 505 nm long pass dichroic with a 450/50 bandpass (425-475 nm) filter in front of the "blue" detector and a 630 nm long pass filter in front of the "red" detector. Cell population showing H342 Bright (H342^Br^) and H342 Low (H342^Lo^) was determined and the ratio of H342^Lo^/H3342^Br ^was calculated to evaluate the dye-efflux capacities of the cells. The gating of H342^Lo ^and H342^Br ^cells was based on a verapamil control. Dead cells were gated out based on positive staining with propidium iodide.

### Determination of cellular growth

Changes in cellular growth, an assessment of proliferation, of parental and colonosphere-derived cells were assessed by 3-(4, 5-dimethylthiazol-2yl)-2, 5-diphenyltetrazolium bromide (MTT) assay as described previously [22]. Briefly, 4000 cells were plated per well in 96-well plates in 200 μl medium. At each time point (0, 24, 48, 72, 96, 120 h), 20 μl MTT solution was added to each well and the plate was incubated for 1 h at 37°C. Medium was then aspirated from each well, and 100 μl DMSO was added. The intensity of the color developed, which is the reflection of number of live cells, was measured at a wavelength of 570 nm. All values were compared to the corresponding controls. All assays were performed with 6 replicates.

### Measurement of Alkaline phosphatase activity

Status of differentiation was determined by measuring the alkaline phosphatase activity using SensoLyte, pNPP alkaline phosphatase assay kit (Anaspec, San Jose, CA) according to the manufacturer's instructions and measuring the absorbance at 405 nm.

### β-catenin siRNA transfection

For the transfection of siRNA into the parental and colonosphere derived cells, Oligofectamine reagent (Invitrogen Corp., Carlsbad, CA) and serum-free Opti-MEM (Invitrogen Corp., Carlsbad, CA) medium was used to prepare transfection complexes according to the manufacturer's instructions. Briefly, single cell suspension of colonospheres was plated in 10 cm tissue culture plates with normal growth medium overnight to achieve 25-30% confluence. Next day the medium was removed, washed twice with serum-free Opti-MEM (Invitrogen Corp., Carlsbad, CA) medium prior to adding the complexes containing non-targeted or β-catenin siRNA (Integrated DNA Technologies Inc., Coralville, IA). After 3 days of transfection, the cells were collected and analyzed for protein expression of β-catenin using Western blot and for colonosphere formation assay using SCM.

### Activation of relative TCF/LEF-Dual Luciferase assay

The activation of transcription factor TCF/LEF was evaluated by using Cignal TCF/LEF reporter assay kit (SA Biosciences, Frederick, MD). The cells were grown to 25-30% confluence as described above and co-transfected with TCF/LEF reporter constructs and either non-targeted or β-catenin siRNAs (Integrated DNA Technologies Inc., Coralville, IA) using SureFECT transfection reagent (SA Biosciences, Frederick, MD) according to manufacturer's instructions. The TCF/LEF reporter used a mixture of an inducible β-catenin-responsive luciferase construct and a constitutively expressing Renilla element (40:1). At the end of 16-24 h incubation period Opti-MEM medium was changed to DMEM 10% FBS for parental cell lines or SCM for colonosphere derived cells. The cells were allowed to grow for another 3 days, collected and analyzed for TCF/LEF activity using a dual-luciferase assay kit (Promega-Biosciences, San Luis Obispo, CA) following the instructions outlined by the manufacturer.

### Overexpression of c-myc gene

Single cell suspension of HCT-116 parental cells was plated in the tissue culture plates to achieve a 90% confluence. Once the 90% confluence is achieved, the adherent cells were transfected using Lipofectamine 2000 and PLUS reagent (Invitrogen Corp., Carlsbad, CA) with plasmid vector-pCMV6-Neo (Origene, Rockville, MD) containing human cDNA clone for c-myc gene or empty plasmid PCMV6-vector in OPTI-MEM medium according to the manufacturer's instruction. After 3 days of transfection, the cells were analyzed for c-myc protein expression by Western blot and re-plated for colonosphere formation assay.

### Statistical analysis

Unless otherwise stated, data are expressed as mean ± SD of six observations. Where applicable, the results were analyzed using analysis of variance followed by Fisher protected least significant differences or Scheffé test. p < 0.01 was designated as the level of significance.

## Competing interests

The authors declare that they have no competing interests.

## Authors' contributions

SSK carried out majority of the experiments and wrote the first draft of the manuscript. He was helped by YY and JN. BBP was involved in the discussion and interpretation of the data. APNM, the principal investigator, was responsible for planning, designing, analysis of the data and overall supervision of the work and final preparation of the manuscript. All authors read and approved the final manuscript.

## Authors' information

Shailender S. Kanwar, Ph.D.: Postdoctoral Research Fellow, Department of Internal Medicine and Veterans Affairs Medical Center, Wayne State University, Detroit, MI 48201, USA. E-mail: sskanwar@gmail.com

Yingjie Yu, M.D., Research Assistant Professor, Department of Internal Medicine and Veterans Affairs Medical Center, Wayne State University, Detroit, MI 48201, USA. E-mail: aa5142@wayne.edu

Jyoti Nautiyal, Ph.D.: Postdoctoral Research Fellow, Department of Internal Medicine and Veterans Affairs Medical Center, Wayne State University, Detroit, MI 48201, USA. E-mail: jyotinautiyal@gmail.com

Bhaumik B. Patel, M.D.: Staff Oncologist and Assistant Professor, Department of Internal Medicine, Veterans Affairs Medical Center and Karmanos Cancer Institute, Wayne State University, Detroit, MI 48201, USA. E-mail: bhaumik.patel@va.gov

Adhip P.N. Majumdar, Ph.D., D.Sc.: Professor and Senior Research Career Scientist, Department of Internal Medicine, Veterans Affairs Medical Center and Karmanos Cancer Institute, Wayne State University, Detroit, MI 48201, USA. E-mail: a.majumdar@wayne.edu
